# Affective and Enjoyment Responses to Short-Term High-Intensity Interval Training with Low-Carbohydrate Diet in Overweight Young Women

**DOI:** 10.3390/nu12020442

**Published:** 2020-02-10

**Authors:** Zhaowei Kong, Mingzhu Hu, Yang Liu, Qingde Shi, Liye Zou, Shengyan Sun, Haifeng Zhang, Jinlei Nie

**Affiliations:** 1 Faculty of Education, University of Macao, Macao 999078, China; zwkong@um.edu.mo (Z.K.); mb74811@um.edu.mo (M.H.); 2 Department of Kinesiology and Program in Neuroscience, Indiana University, Bloomington, IN 47405, USA; YL82@indiana.edu; 3 School of Health Sciences and Sports, Macao Polytechnic Institute, Macao 999078, China; qdshi@ipm.edu.mo (Q.S.); jnie@ipm.edu.mo (J.N.); 4 Exercise and Mental Health Laboratory, School of Psychology, Shenzhen University, Shenzhen 518061, China; liyezou123@gmail.com; 5 Institute of Physical Education, Huzhou University, Huzhou 313000, China; 6 College of Physical Education, Hebei Normal University, Shijiazhuang 050000, China; hbnuzhanghaifeng@sina.com

**Keywords:** overweight, ketogenic diet, intermittent exercise, repeated sprint training, pleasure, adherence

## Abstract

Low-carbohydrate diets (LCs) seem effective on weight reduction and maintenance. However, the affect and enjoyment of exercise during LCs is not clear. The purpose of the present study was to compare the psychological responses to high-intensity interval training (HIIT) and to moderate-intensity continuous training (MICT) during the consumption of a 4-week LC diet in overweight young women. With LCs (~10% carbohydrate, 65%–70% fat, 20%–25% protein), forty-three eligible women (age: 20.9 ± 3.1 years; body weight: 65.8 ± 8.2 kg) were randomly assigned to one of three groups: HIIT (10 sets of 6 s all-out cycling interspersed with 9 s of rest), MICT (30 min cycling at 50%–60% of peak oxygen consumption, V̇O_2peak_) or no-exercise controls (CON). Anthropometric indices and V̇O_2peak_ were measured pre- and post-training. Feeling Scale (FS), Felt Arousal Scale (FAS), Exercise Enjoyment Scale (EES), and Physical Activity Enjoyment Scale (PACES) scores were collected before and immediately after each training session throughout the study. After intervention, all three groups reduced by more than 2.5 kg of body weight whereas both exercise groups improved ~15% V̇O_2peak_. Participants in the HIIT and MICT group exhibited similar affect points as indicated by FS and FAS. Post-exercise enjoyment scores in PACES were lower in HIIT (73–78 points) than MICT (83–87 points) despite similarly positive responses being observed in EES (corresponding to ~4 points of a 7-point scale). Short-term LCs were effective in weight loss and exercise training had an additive improvement on cardiorespiratory fitness. The overweight young women had similar affect valence, arousal levels, and comparable pleasurable feelings to HIIT and MICT with LCs. Furthermore, as indicated by PACES, MICT was more enjoyable which may elicit better adherence, whereas HIIT with LCs seems to be more arduous despite its time-efficiency.

## 1. Introduction

Being overweight or obese is associated with a low level of life quality [1] and a high incidence rate of various diseases such as diabetes, cardiovascular disease, hyperlipidemia and cancer [2,3], which have become severe public issues in numerous countries [4]. It is generally agreed that changing dietary habits helps to lower the rate of obesity. With accumulating evidence showing that low-carbohydrate diets (LCs) are an effective strategy for weight control and reduction [5,6], LCs have received more attention over the years. In addition to a change of diet, previous studies have illustrated benefits of being physically active among overweight and obese individuals [7,8]. Thus, regular exercise is frequently applied as an addition to diets to facilitate weight loss and improve health. Moderate-intensity continuous training (MICT) is a traditional recommendation for improving health in overweight and obese populations [9], yet it requires dedication to an extended amount of time and effort for exercise. Given that perceived lack of time is one of the major barriers for individuals to engage in exercise programs, high-intensity interval training (HIIT) has been advocated as an alternative to MICT, since it is much more time-efficient and has been shown to induce similar health-enhancing adaptations as MICT [10,11].

Although LCs and exercise training can induce weight loss and improve health separately, data related to the effects of combining LCs and exercise training on health promotion are scarce. Given that LC-induced changes in metabolism have been reported to reduce cardiorespiratory fitness (CRF) previously [12,13], our recent study has incorporated additional MICT (30 min/session) or HIIT (2.5 min/session) to a 4-week LC intervention, and compared the effects to a LC control group with no exercise [6]. The physiological results showed significant reductions in body weight and visceral adipose tissue in all three groups, but only the two groups with exercise training reversed the CRF-lowering effects of LC. Of note, the HIIT group induced similar improvements in CRF (~ +3.5 mL·min^−1^·kg^−1^) with 1/12 of the time commitment compared to the MICT group [6]. Thus, from a physiological point of view, it is necessary to combine LCs with exercise training to get additional health benefits, especially for the extremely time-saving HIIT. In addition, participants’ psychological responses to diet and exercise may affect their long-term adherence.

Perceiving affective and enjoyment responses is paramount for adherence to a constructed exercise program in low-activity populations. Although it has been well acknowledged that MICT is associated with positive affective changes [14], the psychological consequences of HIIT are still less clear due to diverse populations and various training protocols. However, despite inconsistent results in affective and enjoyment responses to HIIT compared to MICT [11,15,16,17,18], a recent systematic review and meta-analysis study [11] showed that HIIT may be a viable strategy for obtaining positive psychological responses. In addition, as a particular form of HIIT, the repeated sprint training modality with extremely short exercise duration (≤10 s of work bout at near to maximal intensity) has received increasing attention because of its physiological benefits and time-efficient characteristics [19,20,21,22,23,24]. Furthermore, our previous research revealed that HIIT (i.e., 8 s cycling interspersed with 12 s rest intervals for 20 min) was more enjoyable than moderate-to-vigorous intensity continuous training in overweight and obese individuals [19]. Some recent studies have reported that short sprints with more repetitions were perceived to be more enjoyable than protocols with longer sprints but fewer repetitions in recreational individuals [15,22].

Although combined LCs with exercise training have been proven to bring additional health benefits [6], the affective responses of HIIT and MICT under LC dietary conditions are rarely examined. A recent study indicated that physically active participants engaged in LC for three weeks had more negative affective feelings to regular exercise than those without carbohydrate restriction [25]. The negative feelings brought by LCs may be associated with their effects on exercise performance, as evidenced by several studies of impairments or maintenance in exercise endurance [26,27,28] and reductions in high-intensity exercise performance [29,30,31]. Given that LCs dramatically restrict the energy intake from carbohydrate, which could accelerate the exhaustion of glycogen content to provide energy during exercise, high-intensity exercise performance during LCs may be particularly impaired as carbohydrate is the dominant energy source [31,32]. Additionally, some studies revealed that metabolic adaption to ketosis resulting from LCs may exert positive influences on maintaining or even improving exercise performance [33,34], because a more durable energy source was obtained through the enhanced fat-utilization ability [32] that can help in sparing the limited glycogen storage [35]. Supposedly, an acute bout of HIIT under LC conditions could be more demanding for overweight individuals to perform, and thus they may perceive HIIT to be less enjoyable than an acute bout of MICT under LC conditions since the body still relies heavily on carbohydrate to generate energy. Yet, after physiological adaptation to ketosis, HIIT with LCs may elicit similar or even more preferable psychological responses relative to MICT with LCs.

In order to observe the potential psychological fluctuations after keto-adaption, the affective and enjoyment responses to exercise training were examined before and after each training session across the 4 weeks of intervention in the present study. Furthermore, as an extension of the psychological aspects of our previous study [6], the purpose of this study was to compare the affective and enjoyment responses between the extremely brief HIIT (i.e., 10 × 6-s sprint cycling interspersed with 9-s rest periods, 1 min of hard exercise) and MICT (i.e., continuous cycling at 50%–60% peak oxygen uptake (V̇O_2peak_) for 30 min) regimes across a 4-week LC dietary intervention among overweight young women. The first hypothesis is that, under the condition of LCs, the affective responses to MICT would be more positive than HIIT. The second hypothesis is that the affect and enjoyment responses would fluctuate as participants may perceive HIIT to be more preferable in the later weeks when adapted to ketosis metabolism.

## 2. Methods

### 2.1. Participants

Before recruitment, a power calculation was performed to calculate the sample size using G * Power (Version 3.1), the process of which was the same as that described in our previous study [6]. The results showed that we needed 12 participants for each group. Taking a 25% potential dropout rate into consideration, the goal was to recruit 45 participants in total for three groups.

This study was approved by the Research Ethics Panel of the University of Macau (RC Ref. no. MYRG2017-00199-FED), but was not registered in advance. The inclusion and exclusion criteria, as well as the recruiting and screening procedures, were presented in our previous study [6]. A total of 53 eligible young females, who could be defined as overweight/obese (BMI ≥ 23 kg/m²) based on the cut-off points for the Asian population [36], were recruited after the screening process. All participants provided written informed consent and completed the Physical Activity Readiness Questionnaire (PAR-Q) after research intentions, experimental procedures and requirements were explained in detail. Their menstrual phases for the past 3 months prior to participation were recorded. Then, they were randomly assigned to the LC control group (CON, *n* = 18), the HIIT with LC group (HIIT, *n* = 18), or the MICT with LC group (MICT, *n* = 17). Ten participants who failed to comply with the diet (*n* = 5), participated insufficiently (*n* = 4) or had time conflicts (*n* = 1) were excluded from data analysis. Forty-three participants completed the program and were included in the final data analysis.

### 2.2. Instruments of Affect and Enjoyment

The primary psychological variables were affect and enjoyment. Affective valence was assessed using two self-report scales, the Feeling Scale (FS) [37] and the Felt Arousal Scale (FAS) [38], which have been extensively used to evaluate affective states [39]. Participants were asked to report how they felt at the exact time of measurement. The FS is a 11-point, single item measure of affective valence and the feelings of pleasure/displeasure ranging from −5 (very bad) to +5 (very good). The neutral point is numerically anchored at “0”, the center of this bipolar rating scale. The FAS is a single item, 6-point scale to assess perceived activation or arousal level, rating from low activation (1 point) to high activation (6 points). Enjoyment responses to exercise were evaluated by the Exercise Enjoyment Scale (EES) and the Physical Activity Enjoyment Scale (PACES) [40,41]. The EES is a single item, 7-point scale where the responses state “how much fun you are having regarding the exercise session”. Anchors are given at every integer, ranging from “totally dislike” at 1 to “like very much” at 7. The PACES questionnaire is an 18-item scale (11 items are scored reversely) in which respondents have to rate “how you feel at the moment about the physical activity you have been doing”. The responses range across a 7-point bipolar rating scale (1 point characterized as “feel bored” and 7 points for “feel interested”). The total scores of PACES are between 18 and 126, with higher scores indicating greater enjoyment.

### 2.3. Experimental Procedures

The experimental procedure consisted of a preliminary stage, pre-intervention measures of anthropometric indices and V̇O_2peak_, a 4-week intervention period and outcome measurements after intervention.

During the preliminary stage, all participants received nutrition workshops about how to estimate the amounts and weights of food/drinks, and how to choose appropriate food/drinks during LCs from the dietitian. All participants were required to record their regular diet and daily activities 3 days/week (two weekdays and one weekend day) for two weeks before intervention as a baseline. After the preliminary stage, the participants performed pre-intervention tests of anthropometric indices (weight, height and BMI) and CRF (V̇O_2peak_ test), which were completed 3–5 days before the intervention. During the 4-week intervention period, participants in the CON group only underwent the LC intervention without exercise, whereas participants in the HIIT and MICT groups received 5 sessions/week of supervised HIIT or MICT training alongside the LC intervention. In the post-intervention tests, assessments of anthropometric parameters and CRF were conducted within 3–5 days of the last intervention day, identical to the pre-intervention tests.

### 2.4. Diet Intervention

After the completion of pre-intervention tests, participants in all three groups undertook a 4-week LC intervention, in which fats, proteins, and carbohydrates were aimed to take up approximately 65%, 25% and 10% of their daily energy intakes, respectively. Nutrition workshops on how to estimate the amounts and weights of food/drinks, and how to choose appropriate food/drinks to fulfill the nutrient requirements of LCs were provided to all participants by the dietitian in the preliminary stage. A handout listing the appropriate food/drinks and sample recipes for LCs and matters needing attention during LCs were also given to all participants. They could choose low-carbohydrate foods/drinks based on their own taste, and there was no restriction on the types of fat from saturated and unsaturated sources. Moreover, participants were instructed to accurately fill out 3-day (two weekdays and one weekend day) dietary recording forms for 4 weeks and reported to the laboratory every week with the dietary recording forms to evaluate diet compliance and get follow-up dietary suggestions. To further ensure their compliance with LC, we also provided the participants with reagent strips (UROPAPER, Suzhou First Pharmaceutical Co. Ltd., Suzhou, China) to assess urinary ketones by themselves every day in the morning or after dinner [42]. Food records were analyzed for energy intake and macronutrient contents by the same dietician using the nutrition analysis and management software (NRISM, version 3.1, China). In addition, participants’ queries or questions about the diet approach or the experiment were immediately answered by the researchers throughout the experiment.

### 2.5. Training Intervention

Participants in the CON group only took LC without exercise, whereas participants in the HIIT and MICT groups received 5 sessions/week of supervised HIIT or MICT training at the Kinesiology lab (temperature: 22 °C, humidity: 50%–60%), alongside the LC intervention. The training protocol for participants in the HIIT group consisted of 10 sets of 6s all-out cycling interspersed with 9 s of rest (2.5 min in total). The power output and heart rate (HR; Polar F4M BLK, Kempele, Finland) of each exercise bout were automatically recorded by the pre-installed software (Monark Anaerobic Test Software). Participants in the MICT group participated in a 30-min continuous cycling training session corresponding to 50% of their V̇O_2peak_ for the first 10 sessions and then increasing to 60% of V̇O_2peak_ in the last 10 training sessions. All participants (including the CON group) were required not to participate in any additional exercise and to maintain their habitual routines throughout the intervention period. Their physical activities were assessed by pedometers (Yamax Digi-Walker SW-200, Japan).

During intervention, HR and the Borg’s ratings of perceived exertion (RPE; 0–10 Modified Borg Dyspnoea scale) [43] were recorded in all training sessions, in which the HIIT ratings were obtained before and right after the 5th and 10th exercise bouts, and the MICT ratings were noted down in 5-min intervals over the 30-min continuous exercise. The mean HR (HR_mean_) in the HIIT group was averaged by the four HR values obtained before and after the 5th and 10th exercise bouts, and the HR_mean_ in the MICT group was averaged using the six HR values obtained during each MICT session. The percentage of maximal HR (% HR_max_) was calculated as: HR_mean_ ÷ HR_max_ × 100%, in which the HR_max_ was the maximal HR value obtained in the V̇O_2peak_ test. The scores of FS, FAS and EES were taken before and immediately after each training session, whereas the PACES was completed immediately after the three single-item scales in both training groups. The affect and enjoyment values during the training intervention were ascertained as the average of five training sessions in each week.

### 2.6. Pre- and Post-Intervention Tests

Anthropometric indices (weight, height and BMI) and CRF (V̇O_2peak_ test) were measured 3–5 days before and after the intervention [6]. All pre- and post-intervention tests of anthropometric indices and CRF levels were carried out within the same menstrual phase of the participants (i.e., at the beginning of each participant’s individual follicular stage), and the training sessions were performed between the period of two follicular stages, which were estimated using the self-reported menstrual phase obtained before participation.

### 2.7. Statistical Analysis

Data were analyzed using the PASW software (version 22.0; IBM, Armonk, NY, USA). Primary analysis was performed on a complete case basis. As the dropout rate was substantial, secondary analysis of the main trial with all participants was applied to assess the primary outcomes, including a sensitivity analysis for intention-to-treat to determine the robustness of the results from per protocol population. The missing values were imputed with the expectation-maximization method. The normal distribution of all variables was checked by the Kolmogorov–Smirnov test. Independent sample t-tests were conducted to determine the differences in training parameters between the two exercise groups. Using the baseline values as covariate, analysis of covariance (ANCOVA) was performed to test differences on anthropometric variables and CRF among the three groups. With time (4 time points) and physiological state (pre- and post-exercise) as two within-subject factors and group as the between-subject factor, a three-way repeated measure ANOVA was performed to examine differences in psychological variables of RPE_0–10_, FS, FAS, and EES. A two-way ANOVA (group × time) was used to determine changes in exercise enjoyment (PACES), physical activity and dietary intake from pre- to post-intervention among different groups. Significant effects were subsequently analyzed using the Tukey post-hoc test. Paired sample t-tests were performed to compare differences in affective and enjoyment responses before and after exercise. As effect size (ES) measures of the main and interaction effects, partial *η^2^* was considered small if *η^2^* < 0.01 and large if *η^2^* > 0.14 [44]. Cohen’s d values were calculated to evaluate the ES for the changes of enjoyment scores across different time points, and a *d* value between 0.20–0.49 was classified as a small effect, 0.50–0.79 as a moderate effect, and above 0.80 as a large effect [45]. All tests for statistical significance were standardized at an alpha level of *p* < 0.05, and all results were expressed as mean ± standard deviation.

## 3. Results

### 3.1. The Demographic Characteristics of the Participants

There were no significant differences in anthropometric variables among the three groups (Table 1). The CON, HIIT and MICT groups had a mean body weight of 65.1 ± 7.3 kg, 67.9 ± 10.3 kg, and 64.5 ± 6.4 kg, respectively, corresponding to a BMI of approximately 25.0 kg/m^2^. The baseline CRF levels, as reflected by V̇O_2peak_, were also similar among the three groups (Table 1). A sensitivity analysis confirmed that the baseline characteristics of the per-protocol participants (Table 1) were similar to those of all participants (Table S1).

### 3.2. Dietary Intake, Daily Physical Activity and Training Data

The baseline dietary energy intakes and macronutrient compositions as measured two weeks before the intervention were similar among the three groups. Mean energy intake of the habitual diet was around 2000 kcal in all groups, in which carbohydrate, protein and fat accounted for approximately 46%, 37% and 15% of energy intake, respectively (Table 2). During the intervention, the proportions of energy intake derived from carbohydrate, fat and protein were changed significantly in all groups, with lower carbohydrate consumption, and higher fat and protein consumptions when compared to the habitual diet (*p* < 0.05, data presented in Table 2). The proportion of macronutrients was in line with the planned LC approach in all groups (approximately 10% carbohydrate, 25% protein and 65% fat). In addition, urine ketosis was detected on 96.9% ± 6.0% of the days, suggesting a good dietary compliance among participants. Daily physical activities were monitored using pedometers in the present study, and no differences was found among the CON group, the HIIT group and the MICT group at baseline or any subsequent time points (*p* > 0.05, Table 2).

The total training time of MICT (600 min) was significantly longer than that of HIIT (50 min, *p* < 0.01). In contrast, the exercise intensity was significantly higher in the HIIT group (82% ± 4% HR_max_) compared to that of the MICT group (75% ± 3% HR_max_, *p* < 0.01). Accordingly, the HIIT group produced a greater mean power output (249 ± 34 W) than the MICT group (54 ± 10 W, *p* < 0.01; Table 3). Sensitivity analyses generated identical results in dietary intake, daily physical activity (Table 2 vs. Table S2) and training data (Table 3 vs. Table S3).

### 3.3. Affective and Enjoyment Responses to the Intervention

Participants in the HIIT group reported higher exertion levels (4.5–5.3 points) at all time points when compared to that reported by participants in the MICT group (1.6–2.1 points, *p* < 0.01, Table 4). No within- or between-group differences in FS scores were detected (*p >* 0.05), and the FS scores showed that the participants rated both exercise training regimes between "somewhat pleasurable” and “pleasurable”. As reflected by the FAS scores, the degrees of arousal level were significantly higher after both HIIT and MICT training (*p <* 0.05), with no significant group difference (Table 4). Sensitivity analyses showed identical results in changes of affective and enjoyment responses (Table S4).

**Figure 1:**
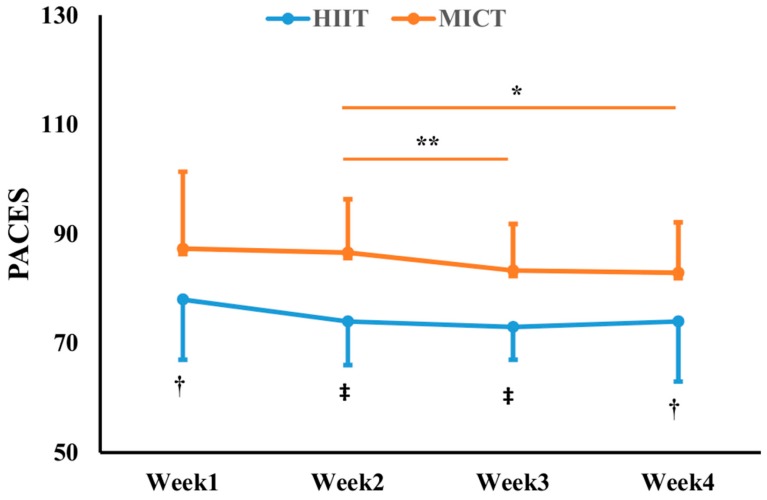
Scores of physical activity enjoyment scale (PACES) during 4-week exercise intervention. HIIT: high-intensity interval training with low-carbohydrate diet; MICT: moderate-intensity continuous training with low-carbohydrate diet. *: *p* < 0.05; **: *p* < 0.01 vs. Week 2; †: *p* < 0.05; ‡: *p* < 0.01 vs. MICT.

There were no significant differences in EES scores before and after exercise in both HIIT and MICT groups, and no group differences were found as well (*p >* 0.05), except for the first week in the MICT group, which showed an increased EES after exercise (Table 4). Regarding the PACES scores, participants in the MICT group had significantly higher PACES scores than those in the HIIT group in all 4 weeks (Figure 1, *F* = 3.422, *p =* 0.033, *η*^2^ = 0.120). Furthermore, there were no significant differences in the PACES scores in the HIIT group during intervention (*p >* 0.05). Within the MICT group, the PACES scores fluctuated across the four training weeks. It displayed that the scores in the second week were higher than those in the third (*p* = 0.009, *d* = 0.36) and fourth weeks (*p* = 0.011, *d* = 0.39), despite the effect sizes being small (Figure 1). A sensitivity analysis confirmed the affect and enjoyment responses to HIIT and MICT during the intervention (Figure 1 vs. Table S5).

## 4. Discussion

Considering the physiological health benefits of combining LC with exercise training [6], it is necessary to examine the participants’ psychological responses to exercise under the LC condition, as it is crucial for long-term adherence. To our knowledge, this is the first study to investigate the psychological responses during HIIT or MICT combined with a LC dietary intervention in the overweight Chinese population. The main findings of the present study were (1) exercise-induced affective states were not significantly different between HIIT and MICT as measured by FS, FAS and EES, and (2) overall, exercise enjoyment in response to MICT was more enjoyable than HIIT as indicated by PACES, despite the PACES scores in the MICT group experiencing a slight decrease in the last two training weeks.

The overall feelings of pleasure towards an exercise, as measured by FS in the present study, is a critical indicator of future adherence. It is generally agreed that high intensity exercises above the ventilatory threshold could evoke displeasure feelings towards exercise, which is known as the “dual-mode” [14]. Negative relations between exercise intensity and affect were observed in several studies, indicating that affect valence was lower in HIIT than MICT [11,18,46]. However, in the present study, despite the fact that the HIIT group had significantly higher exercise intensity and perceived exertion, the FS scores indicated that the participants generally had positive feelings of pleasure (the scores were between “somewhat pleasurable” and “pleasurable”) after both HIIT and MICT exercises, with no significant group difference between these two exercise regimens. The reason that higher exercise intensity and RPE did not reduce the general pleasure responses in HIIT may be explained by the extremely short exercise bouts (10 repetitions of 6 s all-out cycling), since a recent study reported that sprint interval training with shorter exercise bouts was perceived more favorably by obese women [17]. In application of the circumplex model that combines FS and FAS as bipolar scales in interpreting exercise perception [47], the degrees of perceived activation indicated by FAS were also similar in both HIIT and MICT groups, suggesting that both exercise regimens could result in positive psychological states (i.e., excitement).

Enjoyment is another indicator of exercise adherence [11], which involves more specific affective domain directly related to exercise than the affective valence that involves general feeling states [48,49]. Under the condition of maintaining one’s habitual diets, the majority of previous studies conducted either in laboratory or free-living situations reported that the enjoyment of HIIT was equal to, if not greater than, that of MICT in inactive individuals [11,34,40]. Despite the fact that HIIT has a higher metabolic demand, favorable enjoyment responses may still present due to the greater feeling of accomplishment after completion and the less monotonous nature compared with MICT [38]. However, combined with LCs, the present study found similar enjoyment responses to HIIT and MICT while using EES, but higher enjoyment levels after MICT than HIIT while using PACES. The differences in enjoyment responses to exercise may be caused by the different measuring instruments. The EES is a one-item scale, which reflects individuals’ quick or instant responses to acute exercises [37,40]. In contrast, the PACES included 18 bipolar items with high internal consistency [41], involving individuals’ cognition and evaluation processes, and it may be a more comprehensive scale to measure post-exercise enjoyment when compared to EES.

In contrast to our hypothesis, HIIT was reported to be less enjoyable than MICT at all time points as indicated by the PACES scores. The inconsistent findings of exercise enjoyment responses between the present study and previous studies could largely be attributed to differences in the chosen exercise protocols (e.g., work-to-rest ratio, exercise intensity and the total exercise duration) [11,19], traits of the participants (e.g., health and fitness conditions) [50], and more importantly, the dietary changes, given that exercise competency may be impaired by carbohydrate restriction. Consuming a high-fat LC for 1–3 days was reported to be detrimental to exercise performance, because the depletion of liver and muscle glycogen stores reduced carbohydrate oxidation rates [51]. Since HIIT relies majorly on glycogen as the primary fuel source while MICT with lower exercise-intensity can attain more energy from oxidative phosphorylation [32], overweight women may perceive HIIT to be more exhausting and thus less enjoyable than MICT when insufficient carbohydrate was available during LC diet. We hypothesized that perceptions of HIIT may change to be more positive than MICT chronically after keto-adaption, in which the body would have shifted from glucocentric to adipocentric means of energy production, thereby accelerating fat oxidation to break through the constraint of scarcity of endogenous glucose storage, and releasing more energy during exercise [52]. Yet, the unchanged enjoyment responses to HIIT suggested that 4 weeks may be too short to result in keto-adaption. Nonetheless, the PACES scores (˃ 70 points) showed that the participants did not have adverse feelings to HIIT. Therefore, LC combined with HIIT could still be a time-efficient dietary and exercise choice for the overweight/obese population to lose weight and improve CRF.

This study had several strengths, including well-controlled homogeneity of participants, pre-and post-measures in the same phase of the menstrual cycle, and supervision of habitual physical activity and dietary intake, which could interfere with psychological responses to the intervention. Given that mood fluctuates across the menstrual cycle [53], we also carefully selected the training sessions during the period of two follicular stages of the participants in both exercise groups. A no-exercise control group was designed to assure the validity of different exercise regimens while excluding the influence of diet. Based on the fact that the per-protocol analysis of the outcomes was consistent with the intention-to-treat (Tables S1–S5), we were confident of the effects observed. However, several limitations should be mentioned as well. First of all, this study lacked comparison groups of exercise training under normal diet condition, which limited further interpretation of the influences of LCs on the psychological responses to exercise. Second, the present study was only applied for 4 weeks to examine the differences in psychological responses to HIIT and MICT; thus, the long-term effect of keto-adaption on the perception of exercise still remains largely unexplored in overweight and obese populations. Third, there were no indicators to show whether the subjects are adapted to LC or not during the 4-week period. Moreover, the present study only investigated participants’ onsite affect and enjoyment of the two types of training. A follow-up study is needed to investigate willingness to continue such an exercise/dieting style, based on the physical (weight loss and health improvements) and psychological (affect and enjoyment) returns from the intervention.

## 5. Conclusions

Overall, the present study provides initial evidence of the potential impacts of LCs on psychological reactions to two exercise regimens. Given that performing extra exercise during LCs could induce additional benefits on CRF besides weight loss, it is valuable to consider which types of exercises could be long-term adhered to if accompanied with LCs [54]. Our results revealed that the participants’ perceptual responses to both HIIT and MICT were positive in general, but the MICT may be more enjoyable than the HIIT during LCs. Therefore, MICT may be more suitable to be combined with LCs in regard to the long-term engagement by overweight/obese females. Nevertheless, the more time-efficient HIIT protocol may still be worth consideration for those who have insufficient time. Future studies could benefit by examining different exercise protocols in more diverse populations in addition to overweight/obese females.

## Figures and Tables

**Table 1:** Demographic data of the participants at baseline.

	CON (*n* = 15)	HIIT (*n* = 15)	MICT (*n* = 13)
Age (y)	20.9 ± 3.7	20.8 ± 2.7	21.5 ± 3.1
Height (cm)	161.3 ± 4.7	162.9 ± 6.2	160.9 ± 4.3
Weight (kg)	65.1 ± 7.3	67.9 ± 10.3	64.5 ± 6.4
BMI (kg/m^2^)	25.0 ± 2.9	25.5 ± 3.1	24.9 ± 1.9
V̇O_2peak_ (mL/min)	1652 ± 211	1576 ± 278	1506 ± 294
V̇O_2peak_ (mL/min/kg)	24.3 ± 2.6	23.8 ± 2.8	23.4 ± 4.4

Observed values are expressed as means ± standard deviation. CON: no exercise training; HIIT: high-intensity interval training with low-carbohydrate diet; MICT: moderate-intensity continuous training with low-carbohydrate diet; BMI: body mass index; V̇O_2peak_: peak oxygen uptake.

**Table 2:** Energy intake, nutrient proportions, and physical activity before and during intervention.

	Pre_Week 1	Pre_Week 2	Week 1	Week 2	Week 3	Week 4
Energy intake (kcal)						
CON	1824 ± 433	1921 ± 377	1755 ± 587	1573 ± 609	1639 ± 435	1568 ± 439
HIIT	2254 ± 720	2229 ± 444	1793 ± 247	1851 ± 272	1839 ± 287	1855 ± 487
MICT	2176 ± 410	2006 ± 539	2109 ± 487	2059 ± 538	2000 ± 550	1956 ± 431
Carbohydrate (% of energy intake)						
CON	44.7 ± 7.9	46.6 ± 12.5	10.4 ± 6.1	9.4 ± 7.6	9.8 ± 5.6	9.9 ± 9.0
HIIT	47.9 ± 7.3	45.2 ± 9.9	13.4 ± 8.5	10.8 ± 5.7	9.7 ± 6.1	7.6 ± 3.0
MICT	45.1 ± 9.2	46.2 ± 10.3	12.7 ± 8.1	11.1 ± 6.0	8.8 ± 3.0	8.5 ± 3.2
Fat (% of energy intake)						
CON	38.5 ± 6.8	37.1 ± 9.7	67.7 ± 6.9	66.3 ± 7.7	67.9 ± 5.0	68.7 ± 9.3
HIIT	36.3 ± 6.2	37.0 ± 7.7	63.1 ± 9.3	64.3 ± 8.3	68.3 ± 7.8	68.5 ± 10.2
MICT	36.8 ± 8.9	34.9 ± 8.6	63.8 ± 8.4	64.4 ± 7.0	68.2 ± 6.0	68.8 ± 7.6
Protein (% of energy intake)						
CON	15.3 ± 4.1	15.2 ± 5.1	21.9 ± 4.3	24.2 ± 5.1	22.9 ± 5.6	21.6 ± 5.1
HIIT	14.9 ± 2.1	15.3 ± 3.0	23.6 ± 5.4	25.1 ± 7.3	22.0 ± 4.4	23.7 ± 8.1
MICT	14.1 ± 2.7	15.1 ± 2.6	23.4 ± 5.1	24.6 ± 4.6	23.1 ± 5.1	22.9 ± 5.5
Daily physical activities (steps)						
CON	8852 ± 1846	7823 ± 1952	8029 ± 2012	8312 ± 3061	7694 ± 2978	7483 ± 1725
HIIT	7933 ± 3385	7763 ± 2747	8472 ± 1819	8331 ± 2244	9050 ± 1811	8147 ± 2092
MICT	7885 ± 2485	8229 ± 1392	9140 ± 1786	9315 ± 2305	8797 ± 1666	9109 ± 1851

Outcome variables are presented as means (standard deviations). CON: low-carbohydrate diet control group; HIIT: high-intensity interval training with low-carbohydrate diet; MICT: moderate- intensity continuous training with low-carbohydrate diet.

**Table 3:** Training data during 4-week intervention.

	HIIT (*n* = 15)	MICT (*n* = 13)
Total Time (min)	50	600
%HR_max_	82 ± 4	75 ± 3 †
%HRR	68 ± 5	57 ± 5 †
RPE	4 ± 1	1 ± 1 †
Mean power (W)	249 ± 34	54 ± 10 †

Values are presented as means ± standard deviations. HIIT: high-intensity interval training with low-carbohydrate diet; MICT: moderate-intensity continuous training with low-carbohydrate diet; %HR_max_: percentage of training heart rate = training HR/maximal heart rate × 100; %HRR: percentage of heart rate reserve = (training heart rate − rest heart rate)/(maximal heart rate − rest heart rate) × 100; RPE: ratings perceived exertion. Group comparison at †: *p* < 0.01.

**Table 4:** Changes in affect and enjoyment during 4-week exercise intervention.

	HIIT (*n* = 15)				MICT (*n* = 13)			
	W1	W2	W3	W4	W1	W2	W3	W4
RPE0-10								
Pre	0.3 ± 0.3	0.3 ± 0.3	0.5 ± 0.3	0.4 ± 0.3	0.4 ± 0.5	0.5 ± 0.5	0.5 ± 0.5	0.5 ± 0.4
Post	4.5 ± 1.8 *^,^†	4.9 ± 1.4 *^,^†	5.2 ± 1.2 *^,^†	5.3 ± 0.9 *^,^†	1.8 ± 1.0 *	1.6 ± 0.9 *	2.1 ± 1.4 *	2.0 ± 1.1 *
FS								
Pre	1.4 ± 1.5	1.4 ± 1.7	1.4 ± 1.6	1.6 ± 1.9	1.4 ± 1.5	1.4 ± 1.4	1.3 ± 1.4	1.4 ± 1.5
Post	1.3 ± 1.4	1.3 ± 1.6	1.3 ± 1.3	1.4 ± 1.9	1.6 ± 1.5	1.5 ± 1.5	1.5 ± 1.6	1.5 ± 1.6
FAS								
Pre	3.3 ± 1.0	3.2 ± 1.1	3.2 ± 1.1	3.3 ± 1.1	3.5 ± 0.8	3.6 ± 0.8	3.6 ± 0.9	3.6 ± 1.0
Post	3.7 ± 1.0 *	3.9 ± 0.9 *	3.8 ± 1.0 *	3.9 ± 0.9 *	4.2 ± 0.7 *	4.1 ± 0.7 *	4.1 ± 0.8 *	4.0 ± 0.9 *
EES								
Pre	4.0 ± 0.7	4.2 ± 1.2	4.1 ± 1.1	4.2 ± 0.9	3.9 ± 0.6	4.1 ± 0.7	4.0 ± 0.9	3.9 ± 0.9
Post	4.2 ± 0.7	4.1 ± 1.0	4.0 ± 1.0	4.1 ± 0.9	4.2 ± 0.6 *	4.1 ± 0.7	4.0 ± 0.8	3.9 ± 0.9

HIIT: high-intensity interval training with low-carbohydrate diet; MICT: moderate-intensity continuous training with low-carbohydrate diet; RPE: ratings perceived exertion; FS: feeing scale; FAS: felt arousal scale; EES: exercise enjoyment scale. *: *p <* 0.05 vs. Pre; †: *p <* 0.001 vs. MICT.

## Supplementary Materials

The following are available online at https://www.mdpi.com/2072-6643/12/2/442/s1, Table S1: ITT analysis for demographic data at baseline, Table S2: ITT analysis for energy intake, nutrient proportions and daily physical activity before and during intervention, Table S3: ITT analysis for training data during 4-week intervention, Table S4: ITT analysis for changes in affect and enjoyment during 4-week exercise intervention, Table S5: ITT analysis for scores of physical activity enjoyment scale (PACES) during 4-week exercise intervention.
