# Supplementary material for: Affective and Enjoyment Responses to Short-Term High-Intensity Interval Training with Low-Carbohydrate Diet in Overweight Young Women
Source: Nutrients. 2020 Feb 10;12(2):442. doi: 10.3390/nu12020442 (PMC7071177; doi:10.3390/nu12020442)
Supplement: Supplementary file 1 [file nutrients-12-00442-s001.zip › Results from ITT analyses/Table S1.docx]

**Table S1. ITT analysis for demographic data at baseline**

|  | CON (n=18) | | |  | | HIIT (n=18) | | |  | MICT (n=17) | | |
| --- | --- | --- | --- | --- | --- | --- | --- | --- | --- | --- | --- | --- |
| Age (y) | 20.8 | ± 3.6 |  | | 20.9 | | ± 2.6 |  | | 22.0 | ± 3.2 |  |
| Height (cm) | 161.9 | ± 4.6 |  | | 162.1 | | ± 6.0 |  | | 162.2 | ± 5.7 |  |
| Weight (kg) | 64.4 | ± 6.9 |  | | 66.2 | | ± 10.2 |  | | 65.7 | ± 7.4 |  |
| BMI (kg•m^-2^) | 24.6 | ± 2.9 |  | | 25.1 | | ± 3.0 |  | | 24.9 | ± 1.9 |  |
| V̇O_2peak_ (ml•min^-1^) | 1615 | ± 216 |  | | 1559 | | ± 261 |  | | 1587 | ± 304 |  |
| V̇O_2peak_ (ml•min^-1^•kg^-1^) | 25.2 | ± 3.1 |  | | 23.7 | | ± 2.8 |  | | 24.2 | ± 4.7 |  |

Observed values are expressed as means ± standard deviation. CON: no exercise training, HIIT: high-intensity interval training with low-carbohydrate diet, MICT: moderate-intensity continuous training with low-carbohydrate diet, BMI: body mass index, V̇O_2peak_: peak oxygen uptake.
