# Supplementary material for: Affective and Enjoyment Responses to Short-Term High-Intensity Interval Training with Low-Carbohydrate Diet in Overweight Young Women
Source: Nutrients. 2020 Feb 10;12(2):442. doi: 10.3390/nu12020442 (PMC7071177; doi:10.3390/nu12020442)
Supplement: Supplementary file 1 [file nutrients-12-00442-s001.zip › Results from ITT analyses/Table S2.docx]

| **Table S2. ITT analysis for energy intake, nutrient proportions and daily physical activity before and during intervention** | | | | | | |
| --- | --- | --- | --- | --- | --- | --- |
|  | Pre_week 1 | Pre_week 2 | Week 1 | Week 2 | Week 3 | Week 4 |
| Energy intake (kcal) | | |  |  |  |  |
| CON | 1834 ± 401 | 1939 ± 327 | 1772 ± 494 | 1626 ± 527 | 1777 ± 463 | 1704 ± 496 |
| HIIT | 2174 ± 680 | 2167 ± 496 | 1735 ± 286 | 1833 ± 281 | 1890 ± 436 | 1822 ± 463 |
| MICT | 2119± 383 | 2042 ± 473 | 2026 ± 454 | 1884 ± 552 | 1959 ± 485 | 1867 ± 415 |
| Carbohydrate (% of energy intake) | | |  |  |  |  |
| CON | 43.6 ± 7.8 | 44.9 ± 10.8 | 10.2 ± 5.7 | 8.3 ± 6.4 | 8.5 ± 5.0 | 8.3 ± 7.6 |
| HIIT | 48.8 ± 7.9 | 46.5 ± 9.5 | 14.1 ± 8.4 | 11.0 ± 6.2 | 9.8 ± 5.6 | 7.8 ± 3.2 |
| MICT | 45.5 ± 8.5 | 46.0 ± 10.0 | 12.1 ± 7.3 | 11.6 ± 5.4 | 9.4 ± 3.1 | 8.6 ± 3.2 |
| Fat (% of energy intake) | | |  |  |  |  |
| CON | 39.9 ± 7.0 | 38.2 ± 8.5 | 67.5 ± 7.5 | 68.1 ± 6.8 | 68.9 ± 5.2 | 70.5 ± 8.0 |
| HIIT | 35.2 ± 6.5 | 36.3 ± 7.2 | 62.5 ± 9.5 | 64.6 ± 8.7 | 68.4 ± 7.0 | 68.8 ± 9.2 |
| MICT | 37.3 ± 8.1 | 35.0 ± 9.1 | 64.6 ± 7.8 | 65.0 ± 6.3 | 68.6 ± 5.7 | 69.3 ± 6.9 |
| Protein (% of energy intake) | | |  |  |  |  |
| CON | 15.3 ± 3.5 | 15.1 ± 4.3 | 22.5 ± 4.8 | 23.5 ± 4.4 | 23.0 ± 5.3 | 21.3 ± 4.5 |
| HIIT | 15.0 ± 2.7 | 15.2 ± 2.8 | 23.5 ± 5.8 | 24.6 ± 7.0 | 21.8 ± 4.5 | 23.2 ± 7.3 |
| MICT | 14.2 ± 2.7 | 15.1 ± 2.6 | 23.3 ± 5.0 | 23.6 ± 4.4 | 22.0 ± 5.0 | 22.2 ± 5.0 |
| Daily physical activities (steps) | | | | | | |
| CON | 8539 ± 2110 | 7757 ± 2372 | 7704 ± 1984 | 8060 ± 2854 | 7770 ± 2769 | 7414 ±1652 |
| HIIT | 8298 ± 3309 | 7663 ± 2501 | 8215 ±1778 | 8545 ± 2259 | 9255 ± 1772 | 8073 ± 2386 |
| MICT | 8342 ± 2352 | 7904 ± 1938 | 8891 ± 2130 | 9051 ± 2579 | 8397 ± 2053 | 8432 ± 2059 |

Outcome variables are presented as means (standard deviations). CON: low-carbohydrate diet control group, HIIT: high-intensity interval training with low-carbohydrate diet, MICT: moderate- intensity continuous training with low-carbohydrate diet.
