# Supplementary material for: Affective and Enjoyment Responses to Short-Term High-Intensity Interval Training with Low-Carbohydrate Diet in Overweight Young Women
Source: Nutrients. 2020 Feb 10;12(2):442. doi: 10.3390/nu12020442 (PMC7071177; doi:10.3390/nu12020442)
Supplement: Supplementary file 1 [file nutrients-12-00442-s001.zip › Results from ITT analyses/Table S3.docx]

**Table S3. ITT analysis for training data during 4-week intervention**

|  |  | HIIT (n=18) | | MICT (n=17) | |
| --- | --- | --- | --- | --- | --- |
| Total time (min) |  | 50 | | 600 | |
| %HRmax |  | 82 | ± 4 | 76 | ± 4 † |
| %HRR |  | 70 | ± 6 | 60 | ± 7 † |
| RPE |  | 4 | ± 2 | 2 | ± 1 † |
| Mean power (W) |  | 244 | ± 34 | 53 | ± 9 † |

Values are presented as means ± standard deviations. %HRR: percentage of heart rate reserve = submaximal heart rate − rest heart rate/heart rate reserve × 100.

HIIT: high-intensity interval training with low-carbohydrate diet, MICT: moderate- intensity continuous training with low-carbohydrate diet. Group comparison at † *p* < 0.01.
