# Supplementary material for: Affective and Enjoyment Responses to Short-Term High-Intensity Interval Training with Low-Carbohydrate Diet in Overweight Young Women
Source: Nutrients. 2020 Feb 10;12(2):442. doi: 10.3390/nu12020442 (PMC7071177; doi:10.3390/nu12020442)
Supplement: Supplementary file 1 [file nutrients-12-00442-s001.zip › Results from ITT analyses/Table S4.docx]

**Table S4. ITT analysis for changes in affect and enjoyment during 4-week exercise intervention**

|  |  |  |  |  | HIIT | (n=18) |  |  |  |  |  |  |  | MICT | (n=17) |  |  |  |  |
| --- | --- | --- | --- | --- | --- | --- | --- | --- | --- | --- | --- | --- | --- | --- | --- | --- | --- | --- | --- |
|  |  | W1 |  | W2 |  | W3 |  | W4 |  |  | W1 |  | W2 |  | W3 |  | W4 |  |  |
| RPE_0-10_ |  |  |  |  |  |  |  |  |  |  |  |  |  |  |  |  |  |  |  |
| Pre |  | 0.4 | ± 0.3 | 0.4 | ± 0.3 | 0.5 | ± 0.3 | 0.4 | ± 0.3 |  | 0.4 | ± 0.4 | 0.5 | ± 0.4 | 0.4 | ± 0.5 | 0.5 | ± 0.4 |  |
| Post |  | 4.4 | ± 1.9*† | 4.5 | ± 1.5*† | 4.6 | ± 1.5*† | 4.9 | ± 1.3*† |  | 2.2 | ± 1.3* | 2.1 | ± 1.2* | 2.6 | ± 1.5* | 2.6 | ± 1.4* |  |
| FS |  |  |  |  |  |  |  |  |  |  |  |  |  |  |  |  |  |  |  |
| Pre |  | 1.2 | ± 1.4 | 1.4 | ± 1.5 | 1.4 | ± 1.5 | 1.4 | ± 1.6 |  | 1.3 | ± 1.3 | 1.3 | ± 1.2 | 1.3 | ± 1.3 | 1.3 | ± 1.3 |  |
| Post |  | 1.0 | ± 1.5 | 1.2 | ± 1.6 | 1.4 | ± 1.4 | 1.3 | ± 1.7 |  | 1.4 | ± 1.3 | 1.3 | ± 1.3 | 1.4 | ± 1.4 | 1.4 | ± 1.4 |  |
| FAS |  |  |  |  |  |  |  |  |  |  |  |  |  |  |  |  |  |  |  |
| Pre |  | 3.3 | ± 1.0 | 3.2 | ± 1.1 | 3.2 | ± 1.1 | 3.3 | ± 1.1 |  | 3.5 | ± 0.8 | 3.6 | ± 0.8 | 3.6 | ± 0.9 | 3.6 | ± 1.0 |  |
| Post |  | 3.7 | ± 1.0* | 3.9 | ± 0.9* | 3.8 | ± 1.0* | 3.9 | ± 0.9* |  | 4.2 | ± 0.7* | 4.1 | ± 0.7* | 4.1 | ± 0.8* | 4.0 | ± 0.9* |  |
| EES |  |  |  |  |  |  |  |  |  |  |  |  |  |  |  |  |  |  |  |
| Pre |  | 3.7 | ± 0.8 | 3.8 | ± 1.3 | 3.7 | ± 1.2 | 3.7 | ± 1.1 |  | 3.9 | ± 0.5 | 4.1 | ± 0.7 | 3.9 | ± 0.8 | 3.9 | ± 0.8 |  |
| Post |  | 4.3 | ± 0.7* | 4.1 | ± 1.0 | 3.6 | ± 1.1 | 3.7 | ± 1.1 |  | 4.2 | ± 0.5* | 4.1 | ± 0.7 | 3.9 | ± 0.8 | 3.9 | ± 0.8 |  |

RPE: ratings perceived exertion, FS: feeing scale, FAS: felt arousal scale, EES: exercise enjoyment scale, HIIT: high-intensity interval training with low-carbohydrate diet, MICT: moderate-intensity continuous training with low-carbohydrate diet.

* *p* < 0.05 vs. Pre. † *p* < 0.001 vs. MICT.
