# Supplementary material for: Affective and Enjoyment Responses to Short-Term High-Intensity Interval Training with Low-Carbohydrate Diet in Overweight Young Women
Source: Nutrients. 2020 Feb 10;12(2):442. doi: 10.3390/nu12020442 (PMC7071177; doi:10.3390/nu12020442)
Supplement: Supplementary file 1 [file nutrients-12-00442-s001.zip › Results from ITT analyses/Table S5.docx]

**Table S5. ITT analysis for scores of physical activity enjoyment scale (PACES) during 4-week exercise intervention**

| Time | HIIT (n=18) | | MICT (n=17) | |
| --- | --- | --- | --- | --- |
| week1 | 76 | ± 13 | 85 | ± 13 † |
| week2 | 73 | ± 9 | 84 | ± 10 ‡ |
| week3 | 73 | ± 6 | 81 | ± 8 **‡ |
| week4 | 74 | ± 11 | 81 | ± 9 **† |

HIIT: high-intensity interval training with low-carbohydrate diet, MICT: moderate-intensity continuous training with low-carbohydrate diet.

* *p* < 0.05, ** *p* < 0.01 vs. Week 2. † *p* < 0.05, ‡ *p* < 0.01 vs. MICT.
